# In-Plane Cyclic Mechanical Properties of CF/PEEK/TPU Flexible Composite with Zero Poisson Ratio

**DOI:** 10.3390/ma17215302

**Published:** 2024-10-31

**Authors:** Junpeng Gao, Tingting Wang, Hu Xu, Laisheng Han, Baoyan Zhang, Niudong Han, Diantang Zhang

**Affiliations:** 1 Composite Technology Center, AVIC Manufacturing Technology Institute, Beijing 100191, China; 2 Key Laboratary of Ecotext, Jiangnan Univsity, Wuxi 214112, China

**Keywords:** zero Poisson ratio, flexible composite, large in-plane deformation mechanism, cyclic load

## Abstract

This paper presents the in-plane deformation and cyclic mechanical properties of CF/PEEK (Carbon Fiber-Reinforced Polyetheretherketone)-reinforced TPU (thermoplastic polyurethanes) flexible composites with a zero Poisson ratio. A novel CF/PEEK honeycomb reinforcement with a zero Poisson ratio was fabricated by using 3D-printing technology. Then, TPU was bonded in the two sides of the CF/PEEK honeycomb reinforcement. The in-plane deformation ability and cyclic mechanical properties were evaluated. The results show that the zero Poisson ratio flexible composite can achieve a large in-plane plastic deformation of more than 50% and can better maintain the zero Poisson ratio superstructure. By collecting and comparing the mechanical characteristic values of the CF/PEEK flexible composite under a cyclic load, the CF/PEEK flexible composite MH22-t0.6-CT has the best structural stability. The length of the structure was increased by about 12.53%. By studying the deformation mechanism and failure mechanisms of the flexible composites, the in-plane recyclability of the flexible composites was evaluated, which provides the basic research basis for large-scale in-plane deformation composites.

## 1. Introduction

Materials and structures with a zero Poisson ratio have special mechanical properties and are superior to traditional materials in terms of shear capacity, deformation, fracture resistance, energy absorption, and collapsibility [1,2,3]. At present, various new concepts and design methods of zero Poisson ratio materials and structures have been rapidly developed, such as zero Poisson ratio structures obtained through textile structure design [4,5], designing new structures and optimizing them [6,7,8,9], or the in-depth analysis of the deformation mechanism of zero Poisson ratio honeycomb structures [10,11]. However, the existing problems mainly include the following: (1) Although the fabric has a good zero/negative Poisson ratio effect, fabric deformation rate, and temperature adaptability, there is structural locking after a composite with a rubber matrix, and the composite structure cannot reflect a good zero Poisson ratio effect [12]. (2) Although the honeycomb structure has good deformation and designability, it is difficult to achieve large in-plane deformation at present, which cannot meet the needs of adaptive aircraft [13,14,15,16]. Therefore, it is of great theoretical and applicational value to carry out structural designs, deformation simulations, forming preparations, and the application verifications of new zero Poisson ratio reinforced materials in order to provide data support for the development of ultra-large deformation, zero Poisson ratio, and high-load composite materials.

Liu Weidong et al. [1] studied the in-plane elastic modulus of the cellular reinforcement structure of an accordion with zero Poisson ratio. Starting from a theoretical mechanics analysis, considering the influence of internal bending moment, axial force, and shear force, six equivalent moduli (including three elastic moduli and three shear moduli) of an accordion honeycomb were deduced theoretically using the energy method, and the correctness of the equivalent moduli theoretical formula was verified using finite element simulation. Shen Yuan et al. [17] proposed a trapezoidal honeycomb skin support structure applied to the flexible telescopic skin of a deformed wing sandwich. The in-plane expansion characteristics of the support structure were studied using theoretical analysis, and the relationship between the dimensionless equivalent elastic modulus and the three parameters (shape coefficient k, width coefficient t, and height coefficient h) was obtained. The test part was obtained by machining the polyformaldehyde plate with an engraving machine. In addition, by comparing it with the accordion-shaped honeycomb reinforcement structure, it can be found that the trapezoidal honeycomb reinforcement structure has stronger in-plane deformation ability, and its elastic modulus can reach between 0.03% and 10% of the raw material. Olympio et al. [13] designed and studied a hybrid zero Poisson ratio honeycomb reinforcement structure, and the results showed that when the traditional honeycomb was unconstrained in the non-deformation direction, the proposed hybrid honeycomb and accordion honeycomb had a roughly similar in-plane axial stiffness and strain capacity to the traditional honeycomb. However, when the Poisson ratio of the hybrid honeycomb and the accordion honeycomb was zero, the skin was constrained in the non-deformable direction and its axial stiffness did not increase accordingly. In general, the current research on the design of zero Poisson ratio honeycomb structures is relatively sufficient. Starting from theoretical mechanics, the force on the honeycomb’s surface is fully analyzed, the in-plane elastic modulus of various honeycomb structures is accurately predicted [18,19,20], and their mechanical properties are analyzed to determine whether they meet the needs of practical applications [21,22,23]. However, the current materials and preparation processes cannot meet the needs of large deformations in the structural plane of the reinforcement, which limits their application in flexible composite materials. An aryl diazonium reaction has the advantages of simple operation and good controllability.

Fiber-reinforced thermoplastic composites typically have higher stiffness and strength, and carbon fiber is more commonly used in fiber-reinforced thermoplastic composites due to its extremely high specific strength. Although the size of the formed workpiece is small, the material structure is well formed, the strength is high, and the preparation quality is stable through the 3D-printing of short-cut carbon fiber-reinforced polymer, which has become a commonly used preparation process. Boston et al. [16] prepared a multi-stable honeycomb structure using short-cut carbon fiber-reinforced nylon composite materials and evaluated the load-bearing capacity of the honeycomb structure under aerodynamic loads through a combination of experiments and finite element simulation calculations.

Three-dimensional-printing technology, with its simple manufacturing process, high economic benefits, high reliability, environmental friendliness, and extensive material customization capabilities, has gradually become one of the most commonly used technologies in the manufacturing industry. Zaharia et al. [24] prepared lightweight sandwich structures of polylactic acid/polyhydroxyalkanoate-based honeycomb, diamond, and corrugated cores using 3D-printing technology. Heeb et al. [25] used 3D-printing technology to prepare zero Poisson ratio honeycomb reinforcement structures and elastic skins from two different polyurethane materials and assembled them into zero Poisson ratio flexible composite materials using Araldite 2014-2 epoxy resin bonding.

In this paper, zero Poisson ratio flexible composites composed of CF/PEEK and TPU were prepared using 3D-printing technology for the first time. The deformation mechanisms and failure mechanisms of flexible composites were studied using an in-plane uniaxial tensile test and high-speed tensile and compression cyclic load tests, and the in-plane recyclability was evaluated, which provides a basic research basis for large-scale in-plane deformation composites. The controllable forming of honeycomb reinforcement and flexible composite materials with zero Poisson ratio, in-plane deformation >50%, and high fatigue resistance is realized, which provides a reference for the flexible skin selection of aircraft in the future.

## 2. Materials and Methods

### 2.1. Materials

Short carbon fiber (T700)-reinforced polyether ether ketone composites were purchased from INTAMSYS, Shanghai, China, and the short carbon fiber content and length were 10% and 80–100 microns, respectively.

Superelastic thermoplastic rubber material polyurethane (TPU 95A) was purchased from China Weibu 3D Co., Ltd., Shanghai, China.

Epoxy adhesive (DP 420) was purchased from 3M Co. of the USA (Saint Paul, MN, USA).

### 2.2. Preparation of Flexible Composites

**Figure 1:**
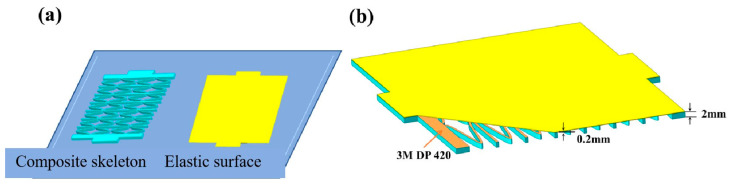
Preparation process of zero Poisson ratio flexible composite. (**a**) Preparation of composite skeleton and elastic surface. (**b**) Schematic diagram of flexible composite structure.

**Figure 2:**
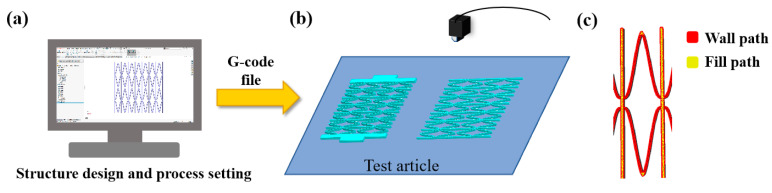
Preparation process of the zero Poisson’s ratio CF/PEEK honeycomb reinforcements. (**a**) Honeycomb reinforcement structural design and print process parameter setting. (**b**) The process of 3D printing. (**c**) The 3D-printing path.

**Figure 3:**
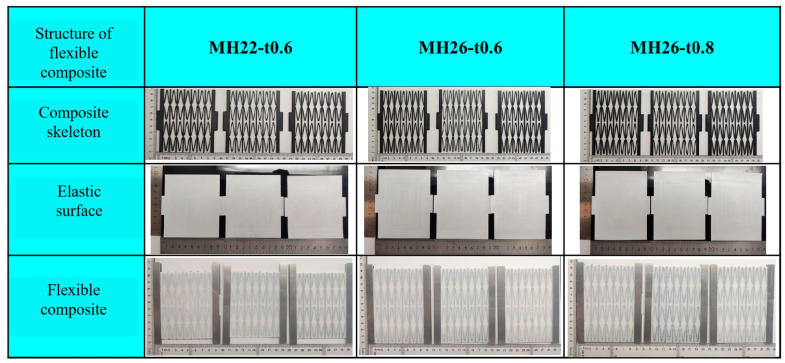
Honeycomb reinforcement, elastic surface, and flexible composite material sample.

The fused deposition (FDM) 3D-printing technology was used to prepare the skeleton and elastic surface of the composite material, respectively, with CF/PEEK and polyurethane as the raw materials. Firstly, establish the CF/PEEK honeycomb reinforcement and TPU elastic skin models separately, and save them as STL format files; secondly, use the CURA 4.1.0 slicing software (Ultimaker, Netherlands) to slice the model and set the 3D-printing process parameters. As shown in Table 1, the generated G-code file was imported into the D260HT 3D printer to produce the CF/PEEK honeycomb reinforcement and TPU elastic skin, as shown in Figure 1a, Figure 2; finally, the composite material skeleton was bonded to the elastic surface to prepare a flexible composite material (Figure 1). Figure 3 shows a flexible composite material specimen.

### 2.3. In-Plane Mechanical Properties Testing and Characterization

The three zero Poisson ratio flexible composites are named MH22-t0.6, MH26-t0.6, and MH26-t0.8, respectively, with suffixes representing the test methods, such as “-T” representing the in-plane tensile test and “-CT” representing the in-plane cyclic loading test.

#### 2.3.1. In-Plane Quasi-Static Mechanical Properties Test

**Figure 4:**
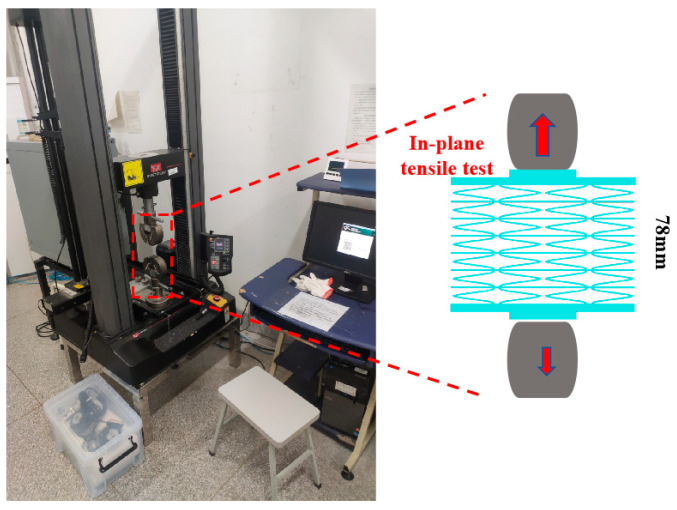
Quasi-static mechanical performance testing.

According to GB/T 1447-2005 “[26] Test Method for Tensile Properties of fiber reinforced Plastics”, the Instron 5967 universal testing machine was used for testing, as shown in Figure 4. The loading rate was 5 mm/min, three samples were selected in each group, and the stress–strain mechanics curve and in-plane tensile modulus were obtained by averaging the results. Poisson’s ratio was recorded and monitored by high-speed cameras. The in-plane tensile elastic modulus *E_t_* is the engineering modulus, calculated by the following formula: the record comparison monitoring of Poisson’s ratio by a high-speed camera. The in-plane tensile elastic modulus *E_t_* is the engineering modulus, calculated by the following formula:

$$E_{t}=\frac{\Delta \sigma }{\Delta \varepsilon }$$

where Δ*σ* is the engineering stress change rate at the elastic deformation stage, while Δ*ε* is the corresponding engineering strain change rate.

#### 2.3.2. High-Speed Tensile Compression Cyclic Load Test

The cyclic drawing speed is set to 234 mm/min. The upper limit of the loading strain is set to 50%, with the lower limit set to 0%. The number of cyclic drawings is set to 200 times, and the interval time between each cyclic drawing is set to 0 seconds.

## 3. Results and Discussion

### 3.1. Composite Skeleton Design

The composite skeleton element utilized in this paper features an internal concave structure and a parallel arrangement of support beams. When subjected to in-plane stretching, the internal concave structure may lead to structural deformation. To enhance the normal stiffness, a parallel configuration of support beams is employed. Table 1 gives the dimensional parameters of the composite skeleton structural unit, whose geometry is determined by the following formula [23]:

$$y=\frac{H}{2}(1-\cos\frac{2\pi x}{L}),x\in [0,L]$$

*L* is the transverse span (mm) of the structural unit, whereas *H* is the longitudinal span (mm) of the structural unit.

Table 2 shows the specific dimensional parameters of the composite skeleton.

When subjected to tensile loading, the shape of the internal bearing plane within the composite skeleton featuring an internal concave structure undergoes changes, while the parallel arrangement of the support beams ensures the composite skeleton exhibits a zero Poisson’s ratio effect. This zero Poisson ratio composite skeleton is capable of achieving uniaxial tensile deformation in the plane without experiencing transverse shrinkage. The composite skeleton constitutes a porous hollow system. The structural parameters of the composite skeleton can be controlled by adjusting both the longitudinal span and the wall thickness of its constituent structural element. At the same time, in order to improve the normal aerodynamic load-bearing capacity of the reinforced structure, more corrugated honeycomb units are arranged horizontally, setting their transverse span to 10 mm and the width of the support beams to 1 mm. Additionally, the structural thickness of the zero Poisson ratio honeycomb reinforcement has been designated as 2 mm.

**Figure 5:**
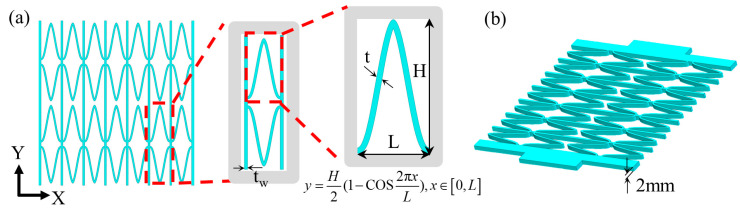
Composite skeleton (**a**) structural dimension parameters and (**b**) three-dimensional model.

According to the previous research results [27], the internal rigidity of a honeycomb reinforcement exhibits an inverse proportionality to the longitudinal span, whereas it is directly proportional to the wall thickness of the honeycomb elements. Moreover, the in-plane rigidity of the honeycomb reinforcement is reduced when the combinations of the longitudinal span and wall thickness of the honeycomb elements are 22 mm and 0.6 mm, 26 mm and 0.6 mm, and 26 mm and 0.8 mm, respectively. It can achieve pure elastic deformation under 50% strain and exhibits excellent in-plane deformation ability. A full-scale model of a composite skeleton was constructed using the commercial modeling software SOLIDWORKS 2022 (3DS Dassault Systemes, France) (Figure 5).

### 3.2. In-Plane Tensile Test Results and Discussion

#### 3.2.1. In-Plane Tensile Mechanical Behavior

**Figure 6:**
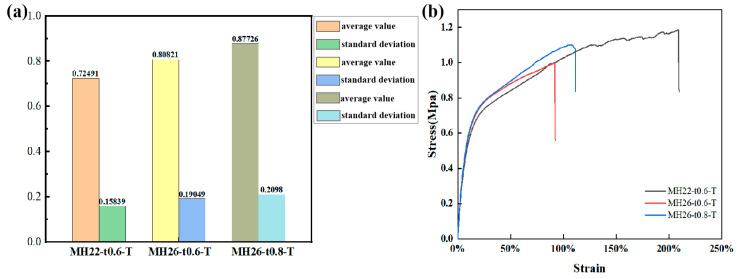
(**a**) Mean–standard deviation graph. (**b**) In-plane tensile test curve of flexible composite with zero Poisson ratio.

Figure 6 shows the mechanical curves resulting from the in-plane tensile tests conducted on three types of flexible composite materials featuring a zero Poisson ratio. The mechanical responses of all these flexible composite materials exhibit consistent pattern and can be roughly divided into three stages: (1) Initial stage and elastic deformation stage: The elastic deformation strain exhibited by the flexible composite materials is minimal, exemplified by the in-plane elastic strain of 1.52% ± 0.12% observed in the MH22-t0.6-T test. During this stage, the slope of the curve is constant, signifying that the stress increases linearly with the increase in strain. It should be noted that the initial portions of the zero Poisson ratio flexible composites nearly coincide, as the in-plane stiffness of the elastic surface is much higher than that of the honeycomb reinforcement, which primarily governs the in-plane mechanical properties of the flexible composites. (2) Plastic deformation stage: In this stage, the slope of the curve ceases to be constant and gradually decreases, resulting in a nonlinear relationship between stress and strain. Although the composite skeleton has a high in-plane elastic strain, the increase in toughness during this period is mainly caused by the plastic changes generated by the highly elastic polyurethane material. (3) Progressive damage stage: For the sample of flexible composite material MH22-t0.6-T, the damage process is quite long and tortuous until the damage and failure finally occur, while for the sample of flexible composite material MH26-t0.6-T and MH26-t0.8-T, the damage and failure stages are relatively brief.

Table 3 records the in-plane tensile modulus of the zero Poisson ratio flexible composites and the composites that have been tested previously. The tensile modulus of the flexible composite is several orders of magnitude higher than that of the composite skeleton. During elastic deformation, the composite skeleton requires minimal driving force, with the majority of the load contributing to the deformation of the elastic surface. Specifically, the tensile modulus of the flexible composite MH26-t0.8-T sample reached 9.51 ± 0.87 MPa, which was about 6.1% higher than that of MH26-t0.6-T, which once again confirmed that the flexible composite controlled its in-plane stiffness mainly through the elastic surface.

#### 3.2.2. In-Plane Tensile Deformation Mechanism and Damage Mechanism

**Figure 7:**
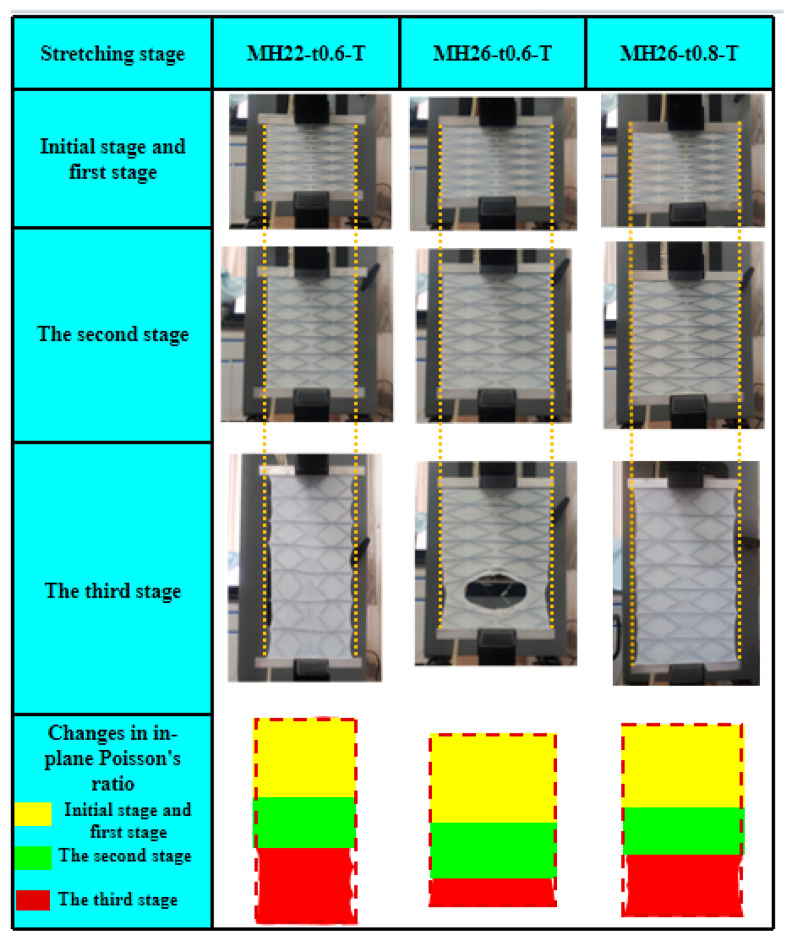
Zero Poisson’s ratio CF/PEEK flexible composite morphology at different tensile stages.

The mechanical curve of the in-plane tensile test shows that the flexible composites first undergo minimal linear deformation and then enter a longer plastic deformation stage as deformation progresses. Figure 7 shows the macroscopic morphology of the in-plane tensile deformation of the flexible composite with zero Poisson ratio. During the initial and elastic deformation stages, it is observable that the composite skeleton undergoes uniform deformation, remaining entirely within the elastic deformation regime without any visible structural damage or failure. In addition, due to the influence of the bonding layer, the elastic surface and the reinforcement remain tightly connected, and the overall structure exhibits a zero Poisson ratio effect. However, as a positive Poisson ratio material, when TPU is subjected to longitudinal loads, the elastic surface layer will produce transverse shrinkage, resulting in a huge shear load on the bond layer, and damage caused by continuous shear load, resulting in damage to the bond layer between the elastic surface layer and the composite skeleton, and finally transition to the progressive damage stage. The reason is that when the elastic surface layer is subjected to tensile load, it is the first to show significant transverse contraction, originating from the center of the structure, and the adhesive layer is subjected to the greatest shear action, leading to the earliest onset of damage and failure. At this time, the flexible composite can no longer maintain the zero Poisson ratio structural characteristics. However, it is noteworthy that despite this transition, the structural strength has not been significantly reduced at this point, but has shifted into a more stable stress plateau. In the third stage, the damage failure of flexible composites mainly occurs between parallel composite skeleton elements. This is mainly because the elastic surface at the failure site is subjected to longitudinal tensile load, while the composite skeleton element drives the elastic surface to produce transverse tensile through bonding, leading to severe stress concentration. The in-plane deformation of the flexible composite at the initial and elastic stages is depicted by a yellow rectangle, whereas the deformation morphology during the plastic deformation stage is indicated by a green rectangle. In the two stages of tensile deformation, the flexible composite can maintain a zero Poisson ratio effect due to the high in-plane deformation capability of the composite skeleton and the good bonding effect between the elastic surface and the composite skeleton elements. The macroscopic morphology of the progressive damage stage is represented by a dark red rectangle. At this stage, the composite structure no longer exhibits the zero Poisson ratio superstructure, and the main damage failure mechanism is the bonding interface layer debonding damage and the elastic surface TPU matrix damage.

### 3.3. Test Results and Discussion of Cyclic Load of High-Speed Tensioning and Compression

**Figure 8:**
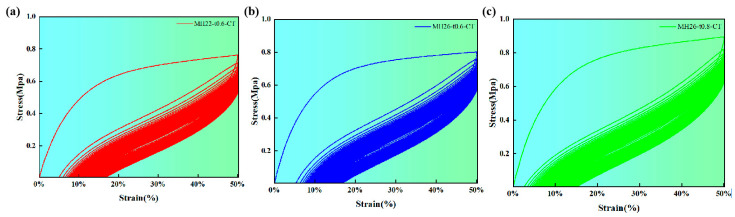
Cyclic loading mechanical curve of flexible composite with zero Poisson ratio. (**a**) MH22-t0.6-CT. (**b**) MH26-t0.6-CT. (**c**) MH26-t0.8-CT.

The mechanical curves of the flexible composite materials with zero Poisson ratio obtained in the cyclic load test are shown in Figure 8. Due to the hysteresis effect, the loading and unloading curves of the mechanical curves do not match, forming a hysteresis loop. This is due to the polymer chain segment of the TPU material which has a relative movement, and the internal friction resistance makes the chain segment movement lag during the loading process. As can be seen from the figure, the hysteresis area obtained by the first loading is the largest, and the energy during the subsequent loading decreases significantly. Furthermore, during the loading process, the unloading curve gradually shifts to the right, with the hysteresis loop area decreasing steadily.

It can be seen from Table 4 that the CF/PEEK flexible composite materials dissipate the most energy under the first loading, which is mainly used for the relative motion of the polymer chain segments and the change in chain segment conformation. Under the action of subsequent loading, the energy dissipated by the flexible composite decreases significantly, and the area of the hysteresis curve decreases significantly. In particular, the flexible composite MH26-t0.6-CT decreases from 0.39 ± 0.01 MJ/m^3^ under the first loading to 0.05 ± 0.01 MJ/m^3^ under the tenth loading, a decrease of 7.8 times. After that, the area of the hysteresis ring decreases and tends to be stable, which indicates that the plastic strain energy of the flexible composite decreases and mainly produces recoverable elastic strain energy.

**Figure 9:**
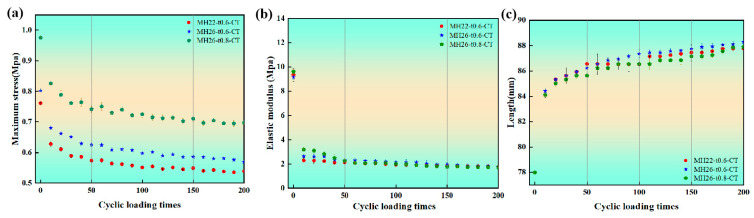
The properties of the zero Poisson ratio flexible composites vary under different cyclic loading times: (**a**) maximum tensile stress; (**b**) elastic modulus; (**c**) sample length.

The maximum tensile stress and tensile modulus changes in the zero Poisson ratio flexible composite under cyclic load are shown in Figure 9a,b. During the initial loading phase, a substantial amount of energy is required to initiate deformation in the flexible composite material, resulting in the highest tensile modulus observed during the first loading cycle. Subsequently, the in-plane stiffness of the flexible composite undergoes a rapid decrease due to the plastic strain of the composite skeleton element and the residual stress of the elastic surface. The tensile modulus of MH22-t0.6-CT is decreased from an initial 9.36 ± 0.53 MPa to 2.30 ± 0.18 MPa. Following this significant drop, the tensile modulus remains relatively stable without undergoing further notable decreases. After the final loading cycle, the tensile modulus of the specimen was 1.75 ± 0.02 MPa. However, in the 40 loading times of MH26-t0.6-CT and MH26-t0.8-CT, the tensile modulus showed stepped attenuation, and then gradually stabilized. Notably, the in-plane stiffness of these three flexible composites exhibited comparable characteristics.

Under the first cyclic load, the tensile stress of each flexible composite is the largest, and the energy driving the deformation of the flexible composites decreases rapidly and tends to be stable due to the generation of residual strain. However, in comparison to the tensile moduli, the maximum tensile stress of MH26-t0.8-CT under cyclic loading is always greater than that of the other two flexible composites. Compared with MH22-t0.6-CT and MH26-t0.6-CT, the maximum stress of the flexible composite MH26-t0.8-CT is increased by about 0.18 MPa~0.22 MPa, which indicates that the influence of the composite’s skeletal structure on its maximum tensile stress is minimal.

Different from the tensile modulus, the maximum tensile stress of MH26-t0.8-CT under cyclic load is always greater than that of the other two flexible composites. This is because the composite skeleton H26-t0.8-T can only achieve 45.8% ± 1.4% in-plane elastic deformation under tensile load. This in-plane deformation capability is inferior to those of the skeletons in the other two flexible composites, which translates into MH26-t0.8-CT requiring greater energy input to attain 50% tensile strain. However, the maximum stress of the composite skeleton H26-t0.8 under tensile load is nearly twice that of the other two composite skeletons, and the maximum stress of the flexible composite MH26-t0.8-CT is increased by 0.18 MPa~0.22 MPa compared with MH22-t0.6-CT and MH26-t0.6-CT. This shows that the influence of the composite skeleton on the maximum tensile stress of the flexible composite is small.

At the beginning of loading, the elastic surface is driven by the load to produce deformation, and the three flexible composites have similar tensile moduli. As the load increases, it drives both the composite skeleton and the elastic surface layer to deform concurrently. However, TPU is a positive Poisson ratio material, so it is accompanied by transverse shrinkage during longitudinal stretching. The binder can effectively connect the composite skeleton and elastic surface layer, but the tensile process will produce a large shear load on the bond layer, which is easy to cause damage and destruction. Although the expansion of the adhesive layer area can effectively alleviate the shear effect, it simultaneously restricts the in-plane shrinkage deformation of the elastic surface. This restriction, in turn, leads to excessive stress concentration within the elastic surface, which is the primary cause of stress relaxation and the potential folding of the material.

Figure 9c shows the change in the structure length of the zero Poisson ratio flexible composite under cyclic loading. At the initial stage of loading, residual strain emerges, which leads to a sharp increase in the length of the flexible composite. It is revealed that the structural length change in the flexible composite is mainly affected by the increase in the residual strain and plastic strain of the elastic surface. In the process of continuous loading, the structural length of each flexible composite increases gradually. The structural length of MH22-t0.6-CT and MH26-t0.8-CT is relatively stable during 60 to 170 loads. Among them, MH26-t0.8-CT has better structural stability than the other two during this period. This is attributed to the fact that MH26-t0.8-CT possesses the largest bonding layer and the largest elastic surface area, which restricts deformation, thereby enhancing the structural stability of the flexible composite during the initial loading process. However, this also means that the relative tensile strength of the remaining elastic surface region, which lacks a bonding layer, should undergo significant changes under tensile load, and the resulting residual should change greatly. As a result, in the final stage of the test, the structural length of MH26-t0.8-CT showed a significant trend of increase.

## 4. Conclusions

The zero Poisson ratio CF/PEEK flexible composite was prepared by 3D-printing technology using CF/PEEK and polyurethane (TPU) as the raw materials. In-plane tensile and high-speed tensile compression cyclic load tests were carried out, the stress–strain curves and mechanical characteristic values were obtained, large deformation and progressive damage evolution studies were carried out, deformation and failure mechanisms were clarified, and the following conclusions were reached:

(1) The in-plane tensile test shows that compared with CF/PEEK honeycomb reinforcement, the in-plane stiffness of the CF/PEEK flexible composite with zero Poisson ratio is dominated by the TPU elastic skin, but it can still achieve a large in-plane plastic deformation of more than 50%, and can better maintain zero Poisson ratio superstructure. The tensile damage failure modes of the CF/PEEK flexible composites are mainly adhesive layer debonding and TPU matrix damage failure. In addition, the bending modulus of the CF/PEEK flexible composite is also improved, and the bending modulus of MH26-t0.8-F reaches 81.18 ± 2.63 MPa, which is about 491.4% higher than that of H26-t0.8-F.
(2) The mechanical properties of the CF/PEEK flexible composite with zero Poisson ratio decrease greatly after the initial loading due to the residual strain generated by the TPU elastic skin under high-speed cyclic loading, and then the decline rate slows down and tends to be stable. By collecting and comparing the mechanical characteristic values of the CF/PEEK flexible composites under cyclic load, the CF/PEEK flexible composite MH22-t0.6-CT has the best structural stability, and the structural length is increased by about 12.53%.

## Figures and Tables

**Table 1:** Preparation process parameters of composite skeleton and elastic surface.

Process Parameter	Composite Skeleton	Elastic Surface
Nozzle temperature/°C	410	210
Base plate temperature/°C	130	40
Ambient temperature/°C	90	25
Print layer thickness	0.1 mm	0.1 mm
Print density	100%	100%

**Table 2:** Specific dimension parameters of composite skeleton.

Composite Skeleton	H (mm)	t (mm)
H22-t0.6	22	0.6
H26-t0.6	26	0.6
H26-t0.8	26	0.8

**Table 3:** Comparison of in-plane tensile modulus between composite skeleton and flexible composite.

Composite Skeleton/Flexible Composite	Tensile Modulus (MPa)
MH22-t0.6-T	9.32 ± 0.21
H22-t0.6-T	0.03 ± 0.01
MH26-t0.6-T	8.96 ± 0.31
H26-t0.6-T	0.01
MH26-t0.8-T	9.51 ± 0.87
H26-t0.8-T	0.05 ± 0.01

**Table 4:** Heat dissipation of flexible composites under cyclic loading.

Composite Skeleton/Flexible Composite	Dissipation Heat (The First)	Dissipation Heat (The 10th)	Dissipation Heat (The 20th)
MH22-t0.6-T	0.1845 MJ/m^3^	0.04315 MJ/m^3^	0.03935 MJ/m^3^
MH26-t0.6-T	0.39315 MJ/m^3^	0.04735 MJ/m^3^	0.04365 MJ/m^3^
MH26-t0.8-T	0.23675 MJ/m^3^	0.05775 MJ/m^3^	0.04975 MJ/m^3^

## Data Availability

Data are contained within the article.

## References

[1] Liu W., Li H. (2018). Cellular equivalent modulus of accordion with zero Poisson’s ratio. Guti Lixue Xuebao/Acta Mech. Solida Sin..

[2] Cheng W., Zhou L., Zhang P., Qiu T. (2015). Design and Analysis of zero Poisson ratio Cruciform hybrid honeycomb and its application in Flexible Composites. Hangkong Xuebao/Acta Aeronaut. Et Astronaut. Sin..

[3] Lu C., Li Y., Dong E., Yang J. (2013). Research on equivalent elastic modulus of honeycomb core with zero Poisson’s ratio. J. Mater. Eng..

[4] Chang Y., Ma P., Jiang G. (2016). Research Progress of Knitting Structures with Negative Poisson Ratio. The 18th Annual Meeting of China Association for Science and Technology. https://www.istis.sh.cn/metadata/resource/did:0a4a393b0cb95eb9c69a2b99912b6ce5.

[5] Lolaki A., Shanbeh M. (2019). Variation of Poisson’s ratio of fabrics woven with helical composite auxetic weft yarns in relation to fabric structural parameters. J. Ind. Text..

[6] Liu K., Cao X., Li Y., Fang D. (2022). Research progress of deformable wing based on chiral superstructure design. Aeronaut. Sci. Technol..

[7] Liu W., Li H., Zhang J., Li H. (2018). Elastic properties of a novel cellular structure with trapezoidal beams. Aerosp. Sci. Technol..

[8] Sun M., Xu Y., Li L. (2016). Structural Stability analysis of variable sweep Angle wing based on ANSYS. Aeronaut. Sci. Technol..

[9] Li T., Li Y., Wu J., Liu M., Wu W. (2016). Structural Parameter Optimization and Selection Analysis of skin Honeycomb Core based on Genetic Algorithm. Mod. Manuf. Eng..

[10] Ai S., Guo Y., Nie X., Chang L. (2021). One-dimensional deformation behavior of honeycomb structures with zero Poisson’s ratio. J. Nanjing Univ. Aeronaut. Astronaut..

[11] Zhang W., Li Z., Jie D., Song N. (2020). Three-dimensional zero-Poisson ratio meso-structure and its macro Structure based on Star Structure. Glob. J. Eng. Sci..

[12] Ma P., Chang Y., Boakye A., Jiang G. (2016). Review on the knitted structures with auxetic effect. J. Text. Inst..

[13] Olympio K., Gandhi F. (2009). Zero Poisson’s Ratio Cellular Honeycombs for Flex Skins Undergoing One-Dimensional Morphing. J. Intell. Mater. Syst. Struct..

[14] Chen J., Shen X., Li J. (2015). Zero Poisson’s ratio flexible skin for potential two-dimensional wing morphing. Aerosp. Sci. Technol..

[15] Liu W., Yang Z., Du S., Li H., Zhang Q. (2022). Theoretical, numerical and experimental study on the in-plane elastic behavior of a 2D chiral cellular structure. Compos. Struct..

[16] Boston D.M., Phillips F.R., Henry T.C., Arrieta A.F. (2022). Spanwise wing morphing using multistable cellular metastructures. Extrem. Mech. Lett..

[17] Shen Y., Ang H., Liu W. (2015). Trapezoidal honeycomb support structure for deformable wing sandwich flexible telescopic skin. Chin. J. Compos. Mater..

[18] Hou W., Shen Y., Jiang K., Wang C. (2022). Study on mechanical properties of carbon fiber honeycomb curved sandwich structure and its application in engine hood. Compos. Struct..

[19] Tripathi L., Behera B. (2021). Review: 3D woven honeycomb composites. J. Mater. Sci..

[20] Gaspar N., Ren X., Smith C., Grima J., Evans K. (2005). Novel honeycombs with auxetic behaviour. Acta Mater..

[21] Zhang L., Liu B., Gu Y., Xu X.H. (2020). Modelling and characterization of mechanical properties of optimized honeycomb structure. Int. J. Mech. Mater. Des..

[22] Ashraf W., Ishak M., MYM Z., Yidris N., Ya’acob A. (2022). Investigation of Mechanical Properties of Honeycomb Sandwich Structure with Kenaf/glass Hybrid Composite Facesheet. J. Nat. Fibers.

[23] Liu W., Zhu H., Zhou S., Bai Y., Wang Y., Zhao C. (2013). In-plane corrugated cosine honeycomb for 1D morphing skin and its application on variable camber wing. Chin. J. Aeronaut..

[24] Zaharia S.M., Enescu L.A., Pop M.A. (2020). Mechanical Performances of Lightweight Sandwich Structures Produced by Material Extrusion-Based Additive Manufacturing. Polymers.

[25] Heeb R., Dicker M., Woods B. (2022). Manufacturing and characterisation of 3D printed thermoplastic morphing skins. Smart Mater. Struct..

[26] Wang F., Zhang G., Chen L. (2010). Study on the Tensile Strength Loss Rate of Glass Fiber 2D Woven Fabric Reinforced Laminates with Ladder Splicing. Appl. Mech. Mater..

[27] Wang T., Gao J., Xu H., Zhang B., Han N., Zhang D. (2023). Finite Element Simulation of In-plane Tensile Mechanical Response of Honeycombs with a Zero Poisson’s Ratio. Aeronaut. Sci. Technol..

